# Dr. Erasmus H. Hall

**Published:** 1917-01

**Authors:** 


**Dr. Erasmus H. Hall,** (not listed in State Directory nor Polk) died at his home in Knowlesville, Dec. 9, aged 85. He had practiced medicine 63 years.

